# Supplementary material for: Bacterial community dynamics during embryonic development of the little skate (Leucoraja erinacea)
Source: Anim Microbiome. 2021 Oct 13;3:72. doi: 10.1186/s42523-021-00136-x (PMC8513177; doi:10.1186/s42523-021-00136-x)
Supplement: Supplementary file 4 — Additional file 4. Figure S1: Internal liquid and egg capsule sampling locations. Egg capsules were windowed to access the developing embryo. Black arrowheads indicate the internal surface of the egg capsule. White arrowhead in A indicates where the transparent, gelatinous internal liquid of closed egg capsules was collected. Open egg capsules were directly drained into microcentrifuge tubes before windowing. Figure S2: ASV rarefaction curves of samples. While number of reads varied between samples, species richness plateaus for each below the maximum sequencing depth. Figure S3: Family-level composition of embryonic and adult skate bacterial communities. Relative abundance of the top ten bacterial families in the classes Alphaproteobacteria (A), Gammaproteobacteria (B), and Bacteroidia (C) in the dataset are shown for each site and timepoint as well as for water and hand controls. Figure S4: Genus-level composition of embryonic and adult skate bacterial communities. Relative abundance of the top ten bacterial genera in the classes Alphaproteobacteria (A), Gammaproteobacteria (B), and Bacteroidia (C) in the dataset are shown for each site and timepoint as well as for water and hand controls. Figure S5: Principal coordinate analysis plots of bacterial communities by tissue. PCoA analysis (Bray-Curtis) plots of PC1 versus PC2 for (A) egg capsule, (B) Internal liquid, (C) gill, and (D) skin samples. Figure S6: Differentially abundant bacterial taxa among skate tissues. LEfSe analysis at P <0.05 and LDA>2. Only differentially abundant tree branches are shown. eggcase: egg capsule, Egillskin: embryonic external gills (stages 16–30) and embryonic skin (stages 16–33), Lgill: internal gill (stage 33-Adult), adultskin: adult skin. Figure S7: Significant taxa comparisons in closed versus open egg capsules. Heatmap of the taxa identified by LEfSe (P<0.05, LDA>2) at the lowest categorized taxonomic level for comparisons of open versus closed egg capsule (red), internal liquid ( [file 42523_2021_136_MOESM4_ESM.docx]

**Supplementary Figure 1: Internal liquid and egg capsule sampling locations.** Egg capsules were windowed to access the developing embryo. Black arrowheads indicate the internal surface of the egg capsule. White arrowhead in A indicates where the transparent, gelatinous internal liquid of closed egg capsules was collected. Open egg capsules were directly drained into microcentrifuge tubes before windowing.

**Supplementary Figure 2: ASV rarefaction curves of samples.** While number of reads varied between samples, species richness plateaus for each below the maximum sequencing depth.


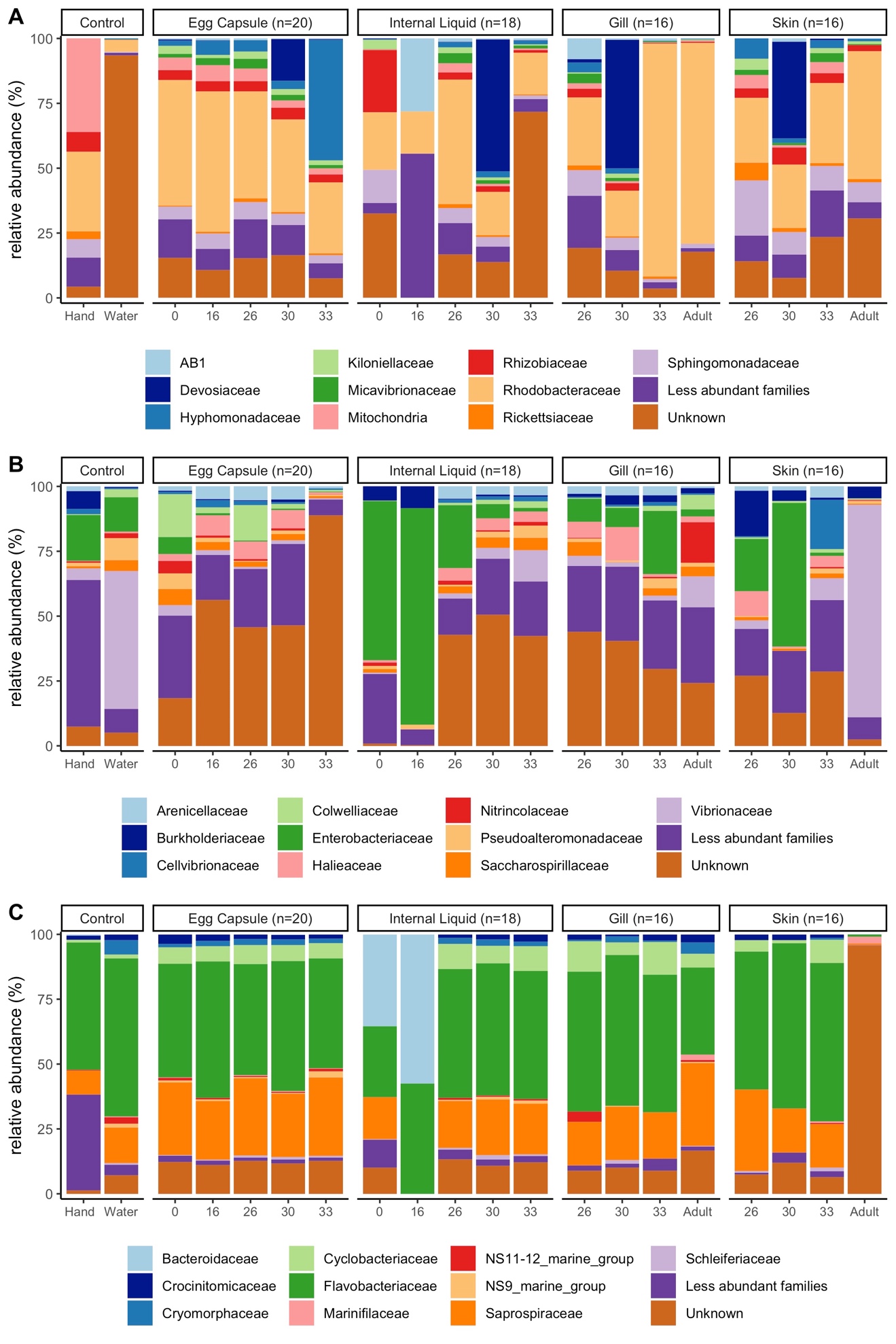


**Supplementary Figure 3: Family-level composition of embryonic and adult skate bacterial communities.** Relative abundance of the top ten bacterial families in the classes *Alphaproteobacteria* (A), *Gammaproteobacteria* (B), and *Bacteroidia* (C) in the dataset are shown for each site and timepoint as well as for water and hand controls. For the controls, n=4 for hand and n=8 for water samples.


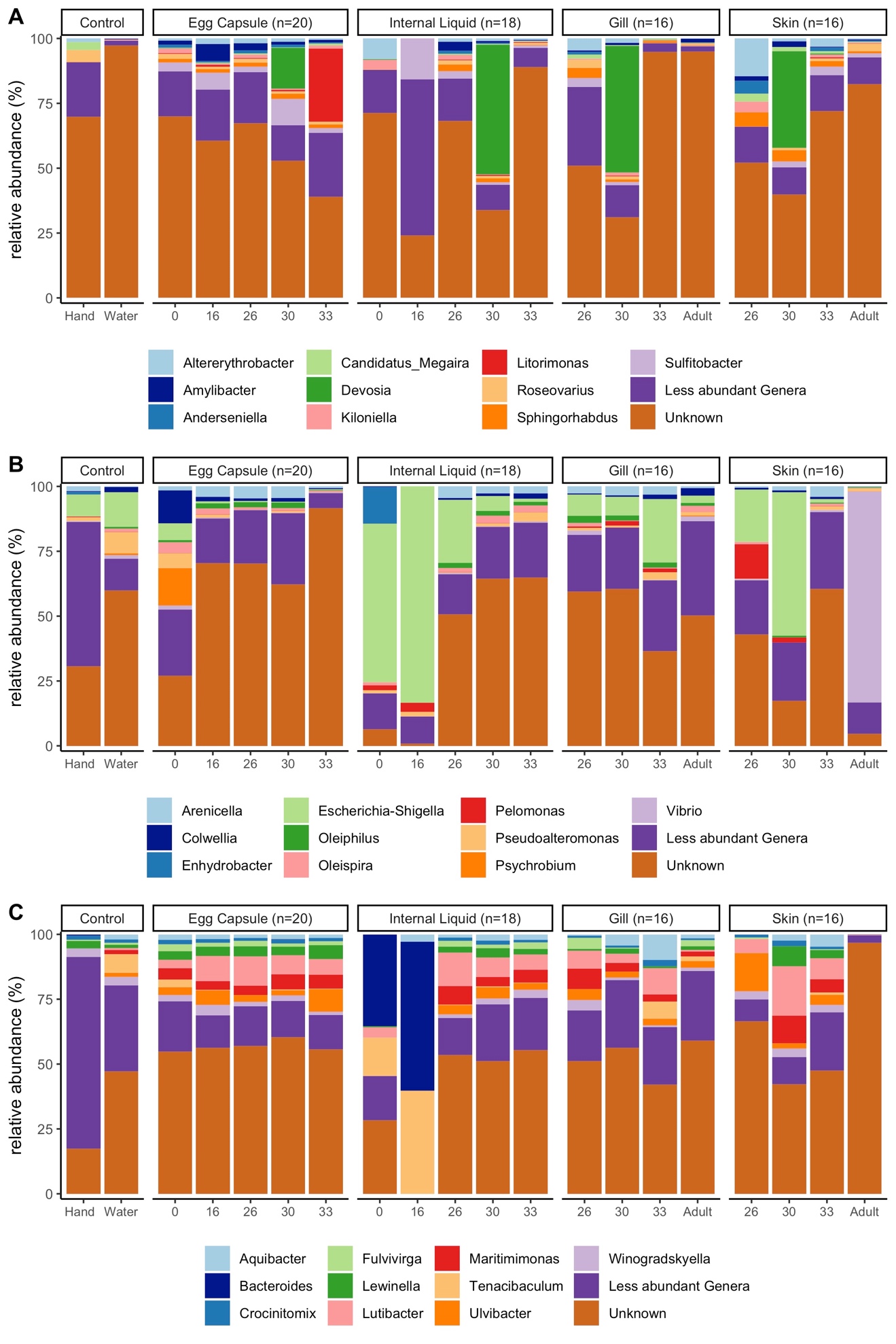


**Supplementary Figure 4: Genus-level composition of embryonic and adult skate bacterial communities.** Relative abundance of the top ten bacterial genera in the classes *Alphaproteobacteria* (A), *Gammaproteobacteria* (B), and *Bacteroidia* (C) in the dataset are shown for each site and timepoint as well as for water and hand controls. For the controls, n=4 for hand and n=8 for water samples.

**Supplementary Figure 5: Principal coordinate analysis plots of bacterial communities by tissue.** PCoA analysis (Bray-Curtis) plots of PC1 versus PC2 for (A) egg capsule, (B) Internal liquid, (C) gill, and (D) skin samples.

**Supplementary Figure 6:** **Differentially abundant bacterial taxa among skate tissues.**

LEfSe analysis at *P* <0.05 and LDA>2. Only differentially abundant tree branches are shown. eggcase: egg capsule, Egillskin: embryonic external gills (stages 16–30) and embryonic skin (stages 16–33), Lgill: internal gill (stage 33-Adult), adultskin: adult skin.

**Supplementary Figure 7: Significant taxa comparisons in closed versus open egg capsules.**

Heatmap of the taxa identified by LEfSe (*P*<0.05, LDA>2) at the lowest categorized taxonomic level for comparisons of open versus closed egg capsule (red), internal liquid (orange) and combined egg capsule and internal liquid (black). Abundances of each sample are shown as Z-scores. Open samples include stage 30 replicates A & D, as well as all stage 33 samples. All other samples come from closed egg capsules.
